# Supplementary material for: Seasonal Shifts in Influenza, Respiratory Syncytial Virus, and Other Respiratory Viruses After the COVID-19 Pandemic: An Eight-Year Retrospective Study in Jalisco, Mexico
Source: Viruses. 2024 Dec 8;16(12):1892. doi: 10.3390/v16121892 (PMC11680140; doi:10.3390/v16121892)
Supplement: Supplementary file 1 [file viruses-16-01892-s001.zip › viruses-3359731-supplementary.pdf]

Table S1. Distribution of respiratory virus cases before and after the COVID-19 pandemic by virus, sex, and age group.

	Influenza							HBoV							VSR						
	Before			After				Before			After				Before			After			
	N	%	$\bar{X}$	N	%	$\bar{X}$	P-value	N	%	$\bar{X}$	N	%	$\bar{X}$	P-value	N	%	$\bar{X}$	N	%	$\bar{X}$	P-value
Female	524	61.21	131	2603	58.25	520.4	<0.001	16	61.54	4.0	6	18.75	1.2	0.431	236	46.46	59.0	543	52.66	108.6	<0.001
Male	332	38.79	83	1866	41.75	373.2		10	38.46	2.5	26	81.25	5.2		272	53.54	68.0	488	47.33	97.6	
Age																					
0-2	97	12.95	24.25	140	4.72	28.00	<0.001	6	30.77	1.50	20	62.50	4.00	0.005	306	60.24	81.5	643	62.43	128.6	<0.001
3-5	53	5.95	13.25	148	3.25	29.60		10	30.77	2.50	2	6.25	0.40		50	9.84	7.5	171	16.60	34.40	
6-14	76	7.82	19.00	574	13.09	114.80		2	7.69	0.50	2	6.25	0.40		22	4.33	5.5	58	5.63	11.60	
15-65	517	60.56	129.25	3279	72.36	655.80		8	30.77	2.00	6	18.75	1.20		56	11.02	14.0	98	9.51	19.60	
>65	113	12.72	28.25	328	6.58	65.60		0	0.00	0.00	2	6.25	0.40		74	14.57	18.5	60	5.83	12.00	
Total	856	100		4469	100			26	100		32	100			508	100		1030	100		

	HEV/HRV							HPIV							HAdV						
	Before			After				Before			After				Before			After			
	N	%	$\bar{X}$	N	%	$\bar{X}$	P-value	N	%	$\bar{X}$	N	%	$\bar{X}$	P-value	N	%	$\bar{X}$	N	%	$\bar{X}$	P-value
Female	110	43.65	27.5	636	52.43	127.2	<0.001	58	31.01	14.5	69	37.80	13.8	0.297	54	61.36	13.5	162	58.27	32.40	<0.001
Male	142	56.35	35.5	577	47.56	115.4		116	68.99	29.0	125	62.20	25		34	38.64	8.5	116	41.73	23.20	
Age																					
0-2	76	30.16	19.00	430	35.45	86.00	<0.001	48	27.59	12.00	84	43.30	16.80	<0.001	38	43.18	9.50	146	52.52	29.20	<0.001
3-5	68	26.98	17.00	203	16.74	40.60		42	24.14	10.50	27	13.92	5.40		40	45.45	10.00	60	21.58	12.00	
6-14	22	8.73	5.55	178	14.67	35.60		14	8.05	3.50	16	8.25	3.20		10	11.36	2.50	11	3.96	2.20	
15-65	76	30.16	19.00	293	24.15	58.60		56	32.18	14.00	35	18.04	7.00		0	0.00	0.00	45	16.19	9.00	
>65	10	3.97	2.55	109	8.99	21.80		14	8.50	3.50	32	16.49	6.40		0	0.00	0.00	16	5.76	3.20	
Total	252	100		1213	100			174	100		194	100			88	100		278	100		

	HCoV-229E						
	Before			After			
	N	%	$\bar{X}$	N	%	$\bar{X}$	P-value
Female	18	75.00	4.50	25	53.19	5.0	0.006
Male	6	25.00	1.50	22	46.81	4.40	
Age							
0-2	2	8.33	0.50	4	8.51	0.80	0.174
3-5	2	8.33	0.50	0	0.00	0.00	
6-14	0	0.00	0.00	2	4.26	0.40	
15-65	20	83.33	5.00	41	87.23	8.20	
>65	0	0.00	0.00	0	0.00	0.00	
Total	24	100		47	100		

	HCoV-OC43						
	Before			After			
	N	%	$\bar{X}$	N	%	$\bar{X}$	P-value
Female	34	73.33	8.5	32	50.79	6.40	0.103
Male	12	26.67	3.0	31	49.2	6.20	
Age							
0-2	0	0.00	0.00	28	44.44	5.60	<0.001
3-5	32	69.57	8.00	2	3.17	0.40	
6-14	2	4.35	0.50	2	3.17	0.40	
15-65	12	26.09	3.00	22	34.92	4.40	
>65	0	0.00	0.00	9	14.29	1.80	
Total	46	100		63	100		

	HCoV-HKU1						
	Before			After			
	N	%	$\bar{X}$	N	%	$\bar{X}$	P-value
Female	8	66.67	2.0	13	43.33	2.60	0.005
Male	4	33.33	1.0	17	56.67	3.40	
Age							
0-2	4	33.33	1.00	5	16.67	1.00	0.375
3-5	0	0.00	0.0	0	0.00	0.00	
6-14	0	0.00	0.0	2	6.67	0.40	
15-65	8	66.67	2.00	20	66.67	4.00	
>65	0	0.00	0.0	3	10.00	0.60	
Total	12	100		30	100		

	HCoV-NL63						
	Before			After			
	N	%	$\bar{X}$	N	%	$\bar{X}$	P-value
Female	2	100.0	0.50	25	43.86	5.0	<0.001
Male	0	0.00	0.00	32	56.14	6.4	
Age							
0-2	0	0.0	0.0	18	31.58	3.60	<0.481
3-5	0	0.0	0.0	1	1.75	0.20	
6-14	0	0.0	0.0	6	10.53	1.20	
15-65	2	100.0	0.50	20	35.09	4.00	
>65	0	0	0.0	12	21.05	2.40	
Total	2	100		57	100		

	hMPV						
	Before			After			
	N	%	$\bar{X}$	N	%	$\bar{X}$	P-value
Female	140	54.47	35	125	53.95	25.00	0.221
Male	117	45.53	29.25	105	46.05	21.00	
Age							
0-2	156	60.70	39.00	98	42.61	19.60	<0.001
3-5	50	19.46	12.50	38	16.52	7.60	
6-14	16	6.23	4.00	14	6.09	2.80	
15-65	29	11.28	7.25	60	26.09	12.00	
>65	6	2.33	1.50	20	8.70	4.00	
Total	257	100		230	100		

The tables include data from influenza viruses (subtypes H1N1, H3N2, and non-subtyped), RSV (Respiratory Syncytial Virus), HPIV (Human Parainfluenza Virus) types 1, 2, 3, and 4, hMPV (Human Metapneumovirus), HCoV (Human Coronavirus) types 229E, NL63, OC43, and HKU1, HAdV (Human Adenovirus), HBoV (Human Bocavirus), and HEV/HRV (Human Enterovirus/Rhinovirus).

Table S2. Complete interrupted time series analysis of respiratory viruses' circulation before, during, and after the COVID-19 pandemic in Jalisco, Mexico.

MAIN MODEL																		
	**Influenza**			**RSV**			**HEV/HRV**			**hMPV**			**HPIV**			**HAdV**		
Variable	β	SE	P-value	β	SE	P-value	β	SE	P-value	β	SE	P-value	β	SE	P-value	β	SE	P-value
Constant (β0)	2.365	0.166	<0.001	0.215	0.193	0.265	-2.821	0.360	<0.001	0.016	0.208	0.937	-0.964	0.254	<0.001	-3.882	0.538	<0.001
Time (β1)	-1.030	0.180	<0.001	0.939	0.191	<0.001	2.988	0.301	0.566	0.405	0.210	0.054	1.049	0.240	<0.001	2.934	0.423	<0.001
Level (β2)																		
2020-2021	-4.266	0.670	<0.001	-6.428	0.629	<0.001	-1.170	0.308	<0.001	-1.200	0.364	0.001	-5.912	1.035	<0.001	-2.268	0.439	<0.001
2022	9.418	0.686	<0.001	5.367	0.658	<0.001	-1.250	0.402	0.002	0.679	0.428	0.112	4.720	1.060	<0.001	-0.331	0.570	0.561
Trend (β3)																		
2020-2021	0.066	0.010	<0.001	0.086	0.009	<0.001	-0.016	0.005	0.002	0.011	0.005	0.043	0.056	0.014	<0.001	-0.006	0.007	0.412
2022	-0.013	0.003	<0.001	-0.024	0.003	<0.001	-0.044	0.004	<0.001	-0.016	0.003	<0.001	-0.017	0.004	<0.001	-0.043	0.005	<0.001

RSV, respiratory syncytial virus. HEV/HRV, human enterovirus/rhinovirus. HPIV, human parainfluenza virus (HPIV), and hMPV (Human Metapneumovirus).

Table S3. Sensitivity analysis of ITS models for respiratory viruses' circulation before, during, and after the COVID-19 pandemic in Jalisco, Mexico.

The sensitivity analyses include the following models:

- **Model 1** accounts for the effect of the strict lockdown period implemented during the early phase of the COVID-19 pandemic.
- **Model 2** uses season instead of year as a categorical variable, providing finer granularity to capture intra-annual variations.
- **Model 3** incorporates the weekly number of COVID-19 cases as an additional variable to assess its concurrent influence on the incidence of other respiratory viruses.

Influenza virus

	Model 1			Model 2			Model 3		
	β	SE	P-value	β	SE	P-value	β	SE	P-value
Constant (β0)	2.365	0.166	<0.001	-	-	-	2.362	0.166	<0.001
Time (β1)	-1.03	0.18	<0.001	-	-	-	-1.023	0.18	<0.001
Intervention (β2)									
2020-2021	-19.809	3.957	<0.001	-	-	-	-3.298	0.702	<0.001
2022	24.961	3.96	<0.001	-	-	-	8.954	0.703	<0.001
Postintervention (β3)									
2020-2021	0.25	0.048	<0.001	-	-	-	0.059	0.01	<0.001
2022	-0.013	0.003	<0.001	-	-	-	-0.018	0.003	<0.001

Model 2 was not computed for Influenza due to the limited number of cases observed during the 2020-2021 season. The small sample size did not allow for reliable seasonal analysis, leading to insufficient statistical power to draw meaningful conclusions for these viruses.

Respiratory syncytial virus

	Model 1			Model 2			Model 3		
	β	SE	P-value	β	SE	P-value	β	SE	P-value
Constant (β0)	0.215	0.193	0.265	-	-	-	0.215	0.193	0.265
Time (β1)	0.939	0.191	<0.001	-	-	-	0.939	0.191	<0.001
Intervention (β2)									
2020-2021	-16.821	1.644	<0.001	-	-	-	-6.57	0.682	<0.001
2022	15.76	1.655	<0.001	-	-	-	5.438	0.679	<0.001
Postintervention (β3)									
2020-2021	0.223	0.021	<0.001	-	-	-	0.088	0.009	<0.001
2022	-0.024	0.003	<0.001	-	-	-	-0.023	0.003	<0.001

Model 2 was not computed for RSV due to the limited number of cases observed during the 2020-2021 season. The small sample size did not allow for reliable seasonal analysis, leading to insufficient statistical power to draw meaningful conclusions for these viruses.

Human metapneumovirus

	Model 1			Model 2			Model 3		
	β	SE	P-value	β	SE	P-value	β	SE	P-value
Constant (β0)	0.016	0.208	0.937	-25.274	96089.738	1	0.005	0.209	0.977
Time (β1)	0.405	0.210	0.054	-15.226	13.058	0.244	0.421	0.210	0.045
Intervention (β2)									
2020-2021	-14.700	2.395	<0.001	14.263	93361.982	1	-0.253	0.457	0.58
2022	14.179	2.405	<0.001	38.755	19805.119	0.998	0.707	0.481	0.141
Postintervention (β3)									
2020-2021	0.183	0.029	<0.001	0.589	0.237	0.013	0.006	0.006	0.286
2022	-0.016	0.003	<0.001	0.130	0.184	0.478	0.026	0.004	<0.001

Human parainfluenza virus

	Model 1			Model 2			Model 3		
	β	SE	P-value	β	SE	P-value	β	SE	P-value
Constant (β0)	-0.964	0.254	<0.001	-25.88	107954.698	1	-0.963	0.254	<0.001
Time (β1)	1.049	0.24	<0.001	4.48	11.534	0.698	1.048	0.24	<0.001
Intervention (β2)									
2020-2021	-5.784	1.066	<0.001	-6.485	105238.753	1	-6.024	1.094	<0.001
2022	4.592	1.09	<0.001	-3.559	22559.596	1	4.805	1.086	<0.001
Postintervention (β3)									
2020-2021	0.054	0.015	<0.001	0.074	0.255	0.771	0.057	0.015	<0.001
2022	-0.017	0.004	<0.001	0.187	0.158	0.236	-0.017	0.004	<0.001

Human enterovirus/rhinovirus

	Model 1			Model 2			Model 3		
	β	SE	P-value	β	SE	P-value	β	SE	P-value
Constant (β0)	-2.821	0.36	<0.001	-25.051	88267.706	1	-2.822	0.36	<0.001
Time (β1)	2.988	0.301	<0.001	-18.394	11.369	0.106	2.989	0.301	<0.001
Intervention (β2)									
2020-2021	-1.08	0.371	0.004	54.66	84685.463	0.999	-1.005	0.34	0.003
2022	-1.34	0.451	0.003	29.523	7883.009	0.997	-1.355	0.408	<0.001
Postintervention (β3)									
2020-2021	-0.018	0.006	0.004	0.126	0.156	0.419	-0.017	0.005	0.001
2022	-0.044	0.004	<0.001	0.02	0.137	0.886	-0.045	0.004	<0.001

Human adenovirus

	Model 1			Model 2			Model 3		
	β	SE	P-value	β	SE	P-value	β	SE	P-value
Constant (β0)	-3.882	0.538	<0.001	-24.157	75189.232	1	-3.885	0.538	<0.001
Time (β1)	2.934	0.423	<0.001	-64.666	19.440	<0.001	2.937	0.423	<0.001
Intervention (β2)									
2020-2021	5.208	0.818	<0.001	116.288	73374.904	0.999	1.301	0.484	0.007
2022	2.607	0.895	0.004	75.250	17177.921	0.997	-0.971	0.583	0.096
Postintervention (β3)									
2020-2021	0.033	0.011	0.005	0.738	0.245	0.003	-0.013	0.007	0.079
2022	-0.04	0.005	<0.001	0.518	0.222	0.02	0.046	0.005	<0.001

Figure S1. Coinfections detected among different respiratory viruses during the study period.

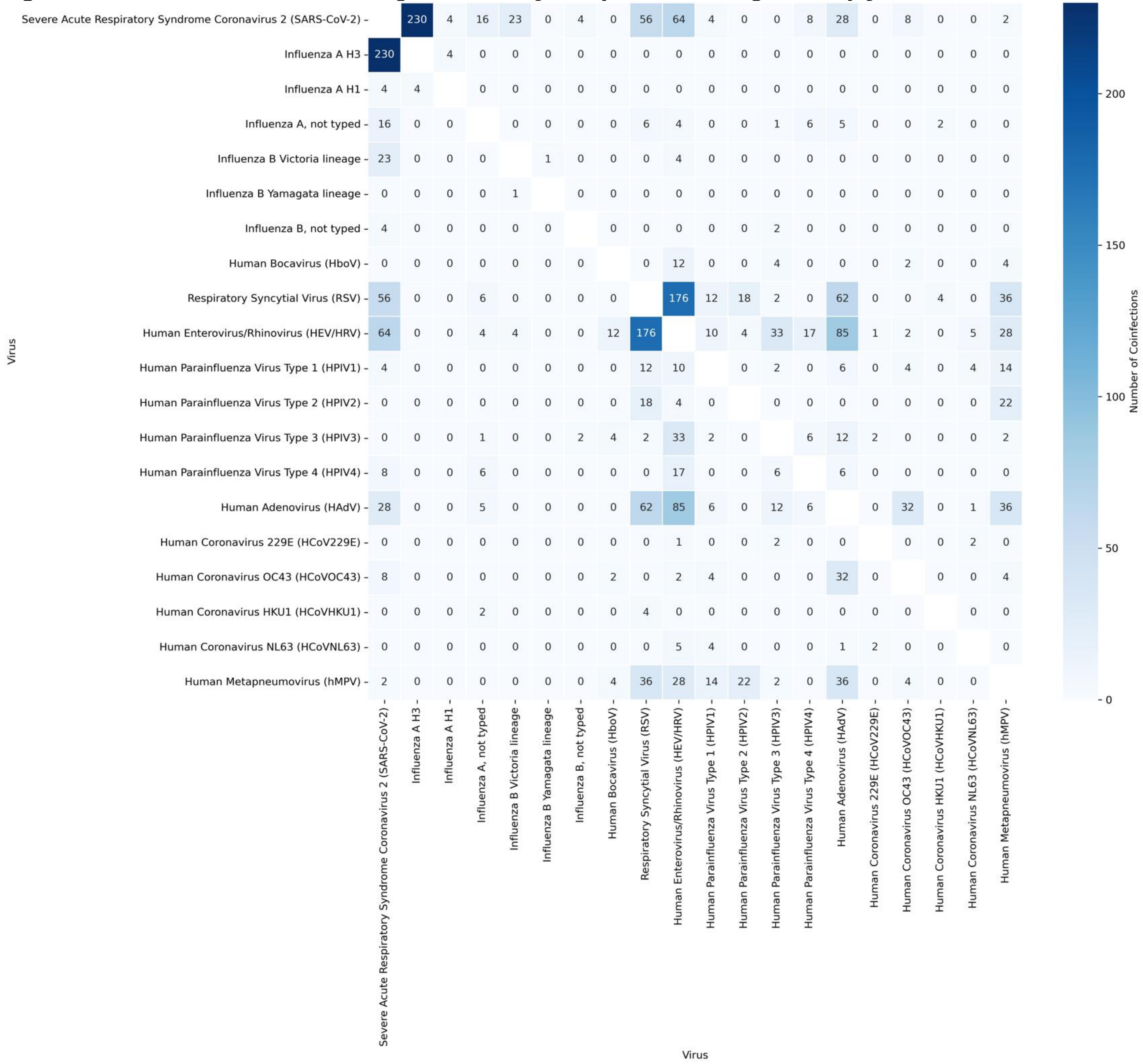

Figure S2 Monthly distribution of positive respiratory virus tests.

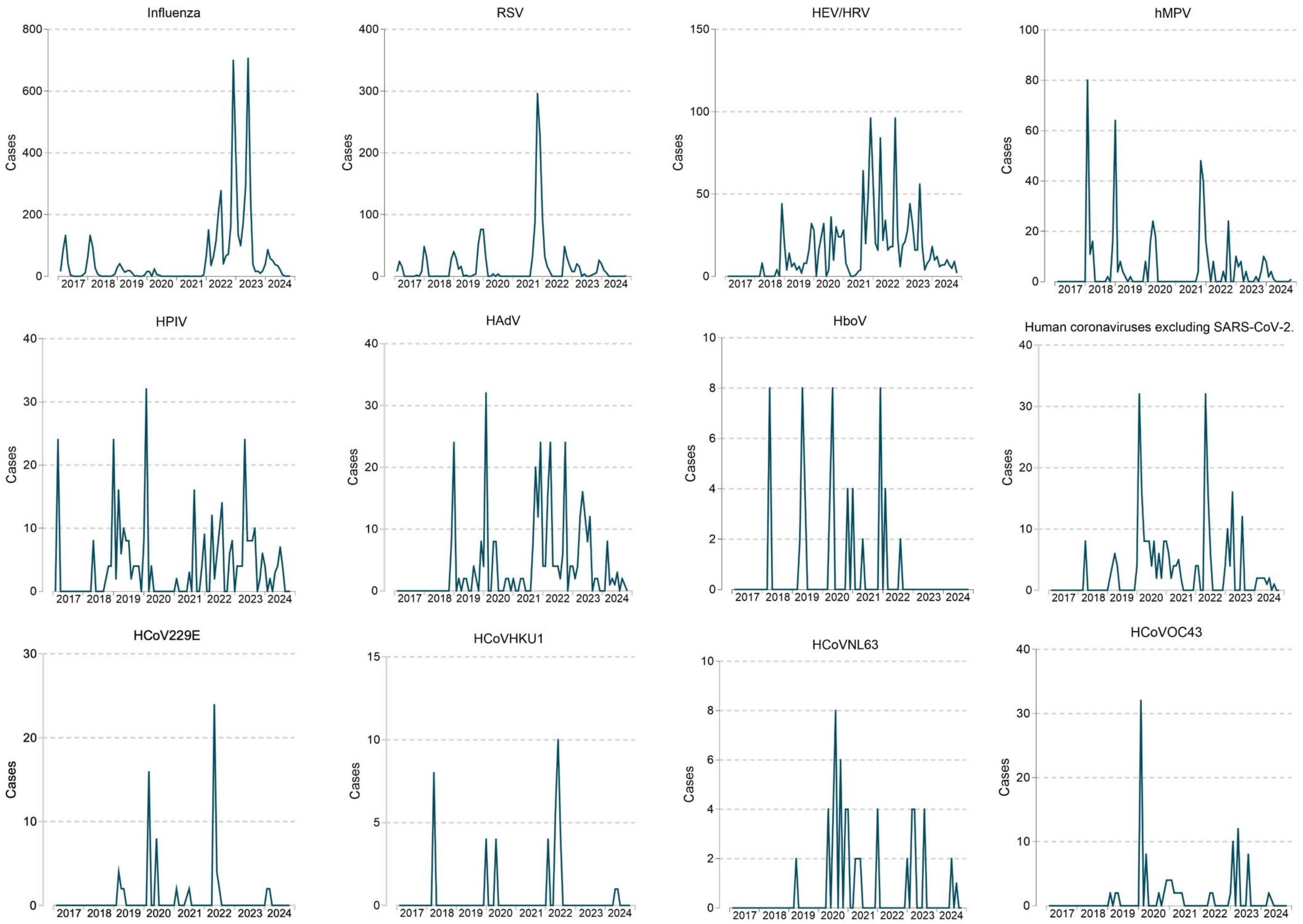

The figure includes data from influenza viruses, RSV (Respiratory Syncytial Virus), HPIV (Human Parainfluenza Virus), hMPV (Human Metapneumovirus), HCoV (Human Coronavirus) types 229E, NL63, OC43, and HKU1, HAdV (Human Adenovirus), HBoV (Human Bocavirus), and HEV/HRV (Human Enterovirus/Rhinovirus).
